# The oncometabolite R-2-hydroxyglutarate dysregulates the differentiation of human mesenchymal stromal cells via inducing DNA hypermethylation

**DOI:** 10.1186/s12885-020-07744-x

**Published:** 2021-01-07

**Authors:** Lizhen Liu, Kaimin Hu, Jingjing Feng, Huafang Wang, Shan Fu, Binsheng Wang, Limengmeng Wang, Yulin Xu, Xiaohong Yu, He Huang

**Affiliations:** 1 Bone Marrow Transplantation Center, The First Affiliated Hospital, Zhejiang University School of Medicine, Hangzhou, 310003 China; 2 Institute of Hematology, Zhejiang University, Hangzhou, China; 3 Zhejiang Province Engineering Laboratory for Stem Cell and Immunity Therapy, Hangzhou, China; 4 Stem Cell Institute, Zhejiang University, 79 Qingchun Road, Hangzhou, Zhejiang Province 310003 P.R. China

**Keywords:** R-2-hydroxyglutarate, IDH mutation, Mesenchymal stromal cells, Differentiation, DNA hypermethylation

## Abstract

**Background:**

Isocitrate dehydrogenase (IDH1/2) gene mutations are the most frequently observed mutations in cartilaginous tumors. The mutant IDH causes elevation in the levels of R-enantiomer of 2-hydroxylglutarate (R-2HG). Mesenchymal stromal cells (MSCs) are reasonable precursor cell candidates of cartilaginous tumors. This study aimed to investigate the effect of oncometabolite R-2HG on MSCs.

**Methods:**

Human bone marrow MSCs treated with or without R-2HG at concentrations 0.1 to 1.5 mM were used for experiments. Cell Counting Kit-8 was used to detect the proliferation of MSCs. To determine the effects of R-2HG on MSC differentiation, cells were cultured in osteogenic, chondrogenic and adipogenic medium. Specific staining approaches were performed and differentiation-related genes were quantified. Furthermore, DNA methylation status was explored by Illumina array-based arrays. Real-time PCR was applied to examine the signaling component mRNAs involved in.

**Results:**

R-2HG showed no influence on the proliferation of human MSCs. R-2HG blocked osteogenic differentiation, whereas promoted adipogenic differentiation of MSCs in a dose-dependent manner. R-2HG inhibited chondrogenic differentiation of MSCs, but increased the expression of genes related to chondrocyte hypertrophy in a lower concentration (1.0 mM). Moreover, R-2HG induced a pronounced DNA hypermethylation state of MSC. R-2HG also improved promotor methylation of lineage-specific genes during osteogenic and chondrogenic differentiation. In addition, R-2HG induced hypermethylation and decreased the mRNA levels of SHH, GLI1and GLI2, indicating Sonic Hedgehog (Shh) signaling inhibition.

**Conclusions:**

The oncometabolite R-2HG dysregulated the chondrogenic and osteogenic differentiation of MSCs possibly via induction of DNA hypermethylation, improving the role of R-2HG in cartilaginous tumor development.

**Supplementary Information:**

The online version contains supplementary material available at 10.1186/s12885-020-07744-x.

## Background

Mutations in isocitrate dehydrogenase (IDH) have been observed in many human malignancies [1]. The initial mutations identified were IDH1 and IDH2 in ~ 80% of intermediate grade gliomas [2] and in ~ 20% of de novo acute myeloid leukemia [3]. Further investigation revealed that frequent IDH mutations were present in both benign and malignant types of cartilaginous tumors, including 71% of conventional chondrosarcomas, 57% of dedifferentiated chondrosarcomas, periosteal chondromas, sporadic central cartilaginous tumors and enchondromas [4–6]. IDH1 mutations cause substitutions at codon R132, while IDH2 mutations affect codon R172 or R140. Recently, IDH2-R172S mutation has been proved to exist in giant cell tumors of bone and osteosarcomas patients [7].

Cytosolic IDH1 and mitochondrial IDH2 are NADP^+^-dependent enzymes that assists in metabolizing isocitrate to α-ketoglutarate (α-KG) in the tricarboxylic acid cycle [1]. Generally, the cancer-associated IDH mutations involves loss of normal catalytic activity of IDH in producing α-KG and gain a neomorphic function of producing R-enantiomer of 2-hydroxylglutarate (R-2HG) [8, 9]. Thus, the IDH1/2 mutant cells are expected to have increased levels of R-2HG, which, are at extremely low concentrations under physiological conditions [10]. Also, the cartilaginous tumors and other neoplasms with IDH1/2 mutations have increased levels of R-2HG [4]. Compelling evidence indicated that IDH1/2 mutation is sufficient to initiate enchondromas and sarcomas in vivo [11, 12]. However, the underlying mechanisms require to investigate further. It is believed that the IDH mutations promote tumorigenesis through putative “oncometabolite” R-2HG accumulation [13]. R-2-HG was also found elevated in colon [14] and breast cancer cells [15] harboring IDH1/2 wild type, despite the R-2-HG levels are lower than IDH mutant. However, the role of R-2-HG in IDH wild type cells in oncogenesis still controversial.

The oncometabolite R-2HG had structural similarities with α-KG, and due to this R-2HG competitively inhibited α-KG dependent enzymes, thereby inducing epigenetic changes including histone modification and DNA hypermethylation [12]. DNA methylation is regarded as an important epigenetic modification that regulates various cellular processes such as differentiation or proliferation. However, dysregulation of it could result in disordered stem cell function or cellular transformation [16]. The epigenetic changes caused mutant IDH protein impaired the differentiation of hematopoietic stem cells and neurogenic precursor cells [13, 17, 18].

Most of the cartilaginous tumors develop from the intramedullary region, and tumor cells were found to be chondrocyte-like in morphology [19]. The clinical findings suggested that bone marrow cells with the capability to differentiate into chondrogenic cells are considered as precursors of this tumor type. Mesenchymal stromal cells (MSCs) could differentiate into chondrogenic, osteogenic and adipocytic lineages, and reside in bone marrow, and are therefore regarded as reasonable precursor cells of cartilaginous tumors [20–22]. Notably, increasing studies have reported that the phenotypic, molecular and gene expressions that run parallelly between the development of chondrosarcoma and the chondrogenic differentiation of MSCs are similar [23, 24]*.* Hence, in this study, the effect of oncometabolite R-2HG on differentiation, proliferation and DNA methylation status of bone marrow MSCs was investigated.

## Methods

### Isolation, culture, and expansion of MSCs

This study was approved by the Ethics Committee of First Affiliated Hospital, School of Medicine, Zhejiang University. Bone marrow (BM) samples were obtained from the healthy adult donors after taking their consent. The mononuclear cells were collected by density gradient centrifugation (Ficoll 1.077 g/mL; Haoyang Biological Manufacture, Co., Ltd., Tianjin, China). The cells were then seeded at a density of 4 × 10^5^ cells/cm^2^ in low-glucose Dulbecco’s modified Eagle’s medium (LG-DMEM; Gibco, Carlsbad, CA, USA) addied with 10% fetal bovine serum (FBS; Gibco) and 100 IU/mL penicillin/streptomycin at 37 °C in a humidified atmosphere containing 5% CO_2_. After 48 h, the non-adherent cells were removed and the medium was replaced every 3 days. The cells after reaching 70–80% confluence were trypsinized and reseeded at a density of 8 × 10^3^ cells/cm^2^. MSCs at passages 3–4 were used in this study.

### Compounds

R-2HG (Sigma-Aldrich, St. Louis, MO, USA) was dissolved in phosphate buffered saline (PBS). The MSCs were treated with 0 to 1.5 mM concentrations of R-2HG.

### Cell proliferation analysis

Proliferation of MSCs was determined using Cell Counting Kit-8 assay (CCK-8; Dojin, Tokyo, Japan). Briefly, the cells were plated at the density of 3000 cells/well in 96-well plates, and then were exposed to R-2HG at a concentration of 0 to 1.5 mM. After culturing at 37 °C in a humidified incubator with 5% CO_2_ for 0, 2, 4, 6, or 8 days, the cells were incubated at 37 °C with 20 μl CCK-8 solution for 2 h, and the absorbance was measured by a multiwell spectrophotometer (Bio-Rad Laboratories, Tokyo, Japan) at 490 nm.

### Flow cytometry assay

MSCs exposed to 0, 1.0, 1.5 mM R-2HG were determined by flow cytometry assay. A total of 5 × 10^5^ cells from single-cell suspensions were incubated for 30 min at room temperature with fluorochrome-conjugated monocolonal antibodies against CD34-PE, CD73-APC, CD90-FITC, CD105-PE (eBioscience, San Diego, CA, USA), CD45-FITC, and HLA-DR-PE-Cy5 (Biolegend, San Diego, CA, USA). After washing with PBS, immunofluorescence analysis was performed by flow cytometry using a FACS Calibur system (Beckman Coulter, Miami, FL, USA) and data were calculated using the FlowJo Software. Appropriate isotype-matched antibodies were used as controls.

### Osteogenic differentiation

MSCs were seeded into 0.1% gelatin coated 6-well plates at a density of 10,000 cells/cm^2^ in LG-DMEM supplemented with 10% FBS. After 2 days, cells were transferred to osteogenic induction medium for 14 days. The medium consists LG-DMEM containing 10% FBS, 10 mM β-glycerophosphate, 0.1 μM dexamethasone and 50 μM ascorbic acid (Sigma-Aldrich). R-2HG at a concentration of 0–1.5 mM was added to the osteogenic induction medium. The medium should be changed for every 3 days. The mineralized areas were revealed using alizarin red staining.

### Chondrogenic differentiation

The cells after reaching 80% confluence were trypsinized, washed, and resuspended in high-glucose DMEM with 1 mM sodium pyruvate (Invitrogen), 0.1 μM dexamethasone (Sigma-Aldrich), 200 μM ascorbic acid (Sigma-Aldrich), 1 × insulin-transferrin-selenium (Invitrogen) and 10 ng/ml transforming growth factor-1 (Peprotech, London, UK). The viable cells were seeded in 15-ml conical tubes at a density of 5 × 10^5^ cells per pellet. Next, the cells were gently allowed to centrifuge to the bottom of the tubes to form compact cell pellets, and then incubated in a humidified atmosphere in 5% CO_2_ at 37 °C. The medium should be changed every 3 days. R-2HG at a concentration of 0–1.5 mM was used for treatment from day 1.

Sections of paraffin-embedded MSCs pellets were processed for immunohistochemistry using rabbit anti-human collagen type II (Abcam, Cambridge, MA, USA). EnVision detection kit (Dako, Carpinteria, CA) was applied to analyze the immunoreactivity of the sections. Non-immune rabbit- IgG antibody was used as the negative control.

### Adipogenic differentiaion

The MSCs were seeded into 6-well plates in a density of 20,000 cells/cm^2^. While cells were grown to confluence, they were transferred to adipogenic induction medium containing LG-DMED and adipogenic stimulatory supplement (Stem Cell Technologies, Hangzhou, China) and the system was cultured for 21 days. The medium was changed every 3 days. R-2HG at a concentration of 0–1.5 mM was added to the adipogenic induction medium. The adipogenic differentiation was mesured by cellular accumulation of large lipid vacuoles that are stained with oil red O (Sigma-Aldrich).

### Real-time quantitative polymerase chain reaction analysis

Messenger RNA (mRNA) expressions of osteogenic (BGLAP, IBSP, LPL, SP7), adipogenic (CEBPA, PPARG, ADIPOQ and FABP4) and chondrogenic differentiation related markers (SOX9, RUNX2, COL2A1 and COL10A1) were quantified using real-time quantitative polymerase chain reaction analysis (RT-PCR). The cultured cell layers or pellets of each group were collected on Day 6 of induction medium incubation. The total RNA was extracted from MSCs using Trizol reagent (Invitrogen) and then was reversely transcribed into complementary DNA (cDNA) by PrimeScript RT reagent Kit (Takara, Tokyo, Japan). Equal amounts of cDNA were used and amplified with SYBR Premix Ex Taq using SYBR Premix Ex Taq (Takara). Every sample was performed in in three independent experiments and all the results were normalized to the levels of glyceraldehydes 3-phosphate dehydrogenase (GAPDH).

The expressions of the components of Sonic Hedgehog signaling pathway including Sonic Hedgehog ligand (SHH), Patched 1 (PTCH1), Smoothened (SMO), and Gli transcription factors (GLI-1, 2 and 3) were quantified by RT-PCR. RNA was prepared from MSCs treated in the absence or presence of 1.0 mM and 1.5 mM R-2HG during osteogenic induction for 6 days.

### Illumina Infinium methylation assay

The changes in DNA methylation of MSCs exposed to R-2HG, and the genome-scale methylation profiles were explored as described previously [25]. MSCs were cultured in proliferation medium in the absence or presence of 1.0 mM R-2HG for 6 days and collected. Bisulfite conversion of genomic DNA was prepared using EZ DNA methylation Kit (Zymo Research, D5002, USA). A total amount of 500 ng of DNA was bisulfite converted and subsequently processed for hybridization onto an Infinium Human Methylation 450 Bead Array (Illumina, San Diego, CA, USA) under the manufacturer’s instructions. This array can interrogate 27,578 CpG dinucleotides encompassing 14,495 genes. In brief, the DNA was mixed with bisulfite, and the nonmethylated C nucleotides were converted to U (T), whereas the methylated C nucleotides remained to be unaffected. Subsequently the bisulfite-treated DNA was amplified, fragmented, and hybridized to locus-specific oligonucleotides on the BeadArray. C or T nucleotides were detected by fluorescence signaling in order to obtain the single-nucleotide extension of the DNA fragments. The results were interpreted as a ratio (β value) of methylated signal (C) when compared with the sum of methylated and unmethylated signal (C-T) for each locus, where 0 was regarded as fully unmethylated DNA and 1 as fully methylated DNA.

To investigate the methylation state of MSCs during osteogenic and chondrogenic differentiation, the cultured cell layers or pellets of MSCs treated either in absence or presence of 1.0 mM R-2HG were collected on Day 6 of induction medium incubation. Bisulfite conversion of genomic DNA was prepared using EZ DNA methylation Kit (Zymo Research, D5002, USA). A total amount of 500 ng of DNA was bisulfite converted and subsequently processed for hybridization onto an Infinium Human Methylation 850 Bead Array (Illumina, San Diego, CA, USA) under the manufacturer’s instructions. Methylation analysis was performed using the R/Bioconductor package Minfi. Methylated CpG sites in promotor region of related genes (BGLAP, IBSP, LPL, SP7, SOX9, RUNX2, COL2A1, COL10A1, SHH, PTCH1, SMO, GLI-1, 2 and 3) were analyzed from the array-based data.

### Heat maps

The heat maps were designed by Mev software. The Euclidean distance within the two groups of samples was calculated using the average linkage measure [the mean of all pair-wise distances (linkages) between the members of the two concerned groups]. Gene annotation and enrichment analyses were performed by KEGG databases using the DAVID Bioinformatics Resources (http://david.abcc.ncifcrf.gov/) interfaces and WebGestalt (http://bioinfo.vanderbilt.edu/webgestalt/), respectively.

### Gene pathway analysis

To determine the biological processes enriched within genes of differential methylation in the comparisons, we uploaded the gene lists into the Ingenuity Pathway Analysis (IPA; Ingenuity Systems, Redwood City, CA, USA). Each gene symbol was linked to its corresponding gene object in the Ingenuity Pathways Knowledge Base. Then the IPA integrates the genes and molecules that share part of the same biological functions or regulatory networks interacting together. The over-represented cellular and molecular functions were ranked according to the calculated *P*-value.

### Statistical analysis

The results are expressed as mean ± standard error (SE), each performed in duplicates. Statistical analysis was performed by analysis of variance (ANOVA). All analyses used SPSS software (Paris, France). A *p*-value of < 0.05 was considered significant.

## Results

### R-2HG did not influence the proliferation and phenotype of human MSCs

The effect of R-2HG on the proliferation of MSCs was examined by CCK-8 assay. As shown in Supplement Fig. 1A, R-2HG showed no affect on the proliferation of MSCs at concentrations 0.1 mM, 0.5 mM, 1 mM or 1.5 mM.

The expression of surface antigens of MSCs was analyzed using flow cytometry. As shown in Supplement Fig. 1B, R-2HG had no influence on the immunophenotype of MSCs, shown as positive for CD105, CD90 and CD73 and negative for CD34, CD45 and HLA-DR.

### R-2HG inhibits osteogenic differentiation of MSCs

**Fig. 1:**
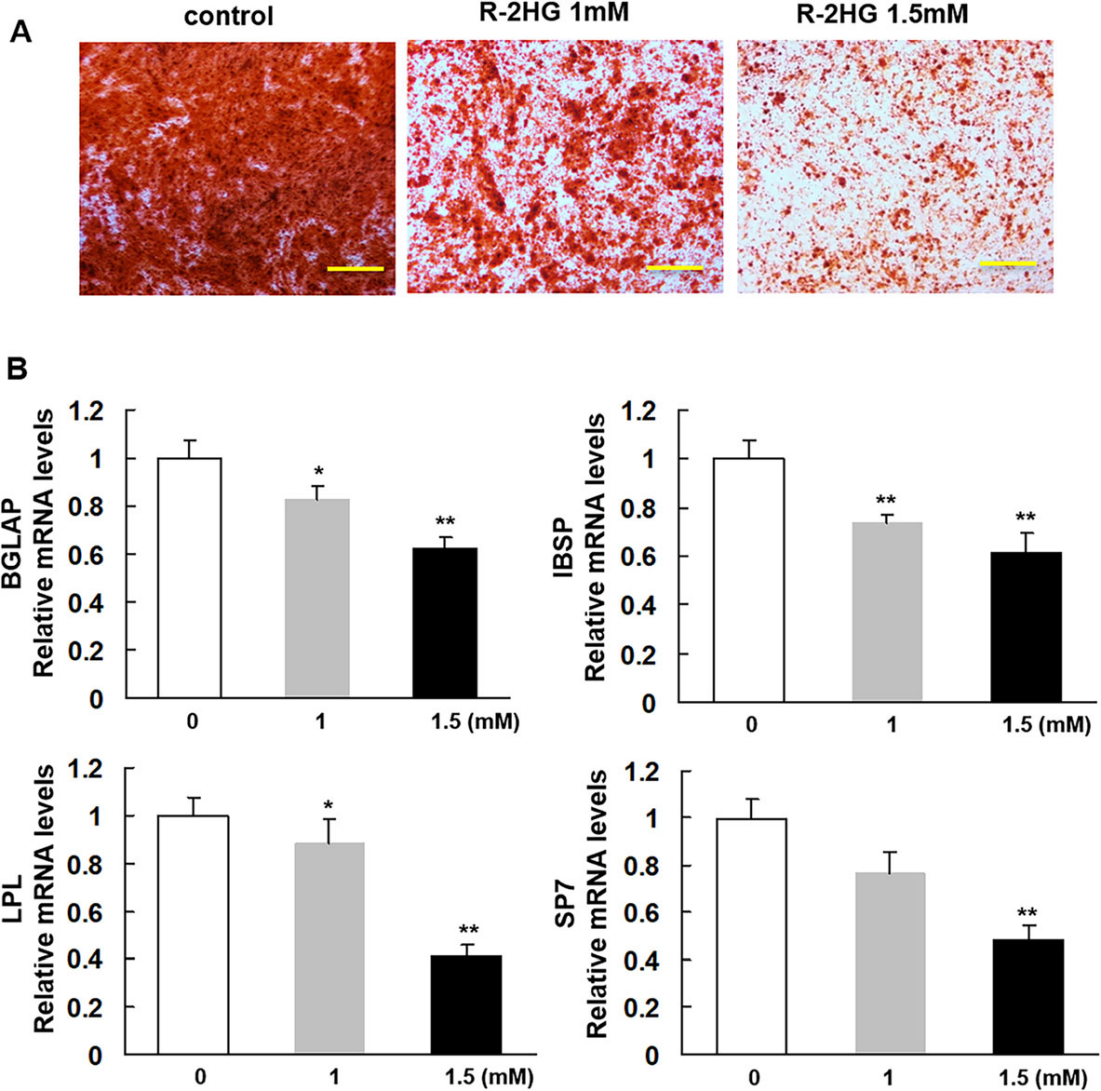
Osteogenic differentiation of mesenchymal stromal cells (MSCs) in the presence of R-2HG. **a**. MSCs were grown in osteo-inductive medium for 14 days in the absence or presence of R-2HG (1, 1.5 mM). Mineralization was assessed by Alizarin red staining. Scale bar = 100 μM (**b**): Quantitative results of expression levels of differentiation-related genes. * *P* < 0.05 vs the group of MSC in absence of R-2HG. ** *P* < 0.01 vs the group of MSC in absence of R-2HG

Osteogenic differentiation in MSCs in the presence of R-2HG (1, 1.5 mM) showed a dose dependent impaired calcification when compared to MSCs in the absence of R-2HG. Alizarin red staining revealed a low extent of mineralization with less detectable bone nodules in R-2HG treated MSCs when compared to those in controls (Fig. 1a). To further investigate the effects of R-2HG on MSC differentiation, we analyzed the relative mRNA expression levels of osteoblast-specific transcription factors (LPL and SP7) and osteoblastic markers (IBSP and BGLAP). The results showed that R-2HG reduced the expression level of both early (LPL and IBSP) and late (Osterix and BGLAP) osteoblast differentiation-related genes significantly, which is consistent with the results in the functional assays (Fig. 1b).

### R-2HG inhibits chondrogenic differentiation of MSCs, but promotes the expression of genes related to chondrocyte hypertrophy

**Fig. 2:**
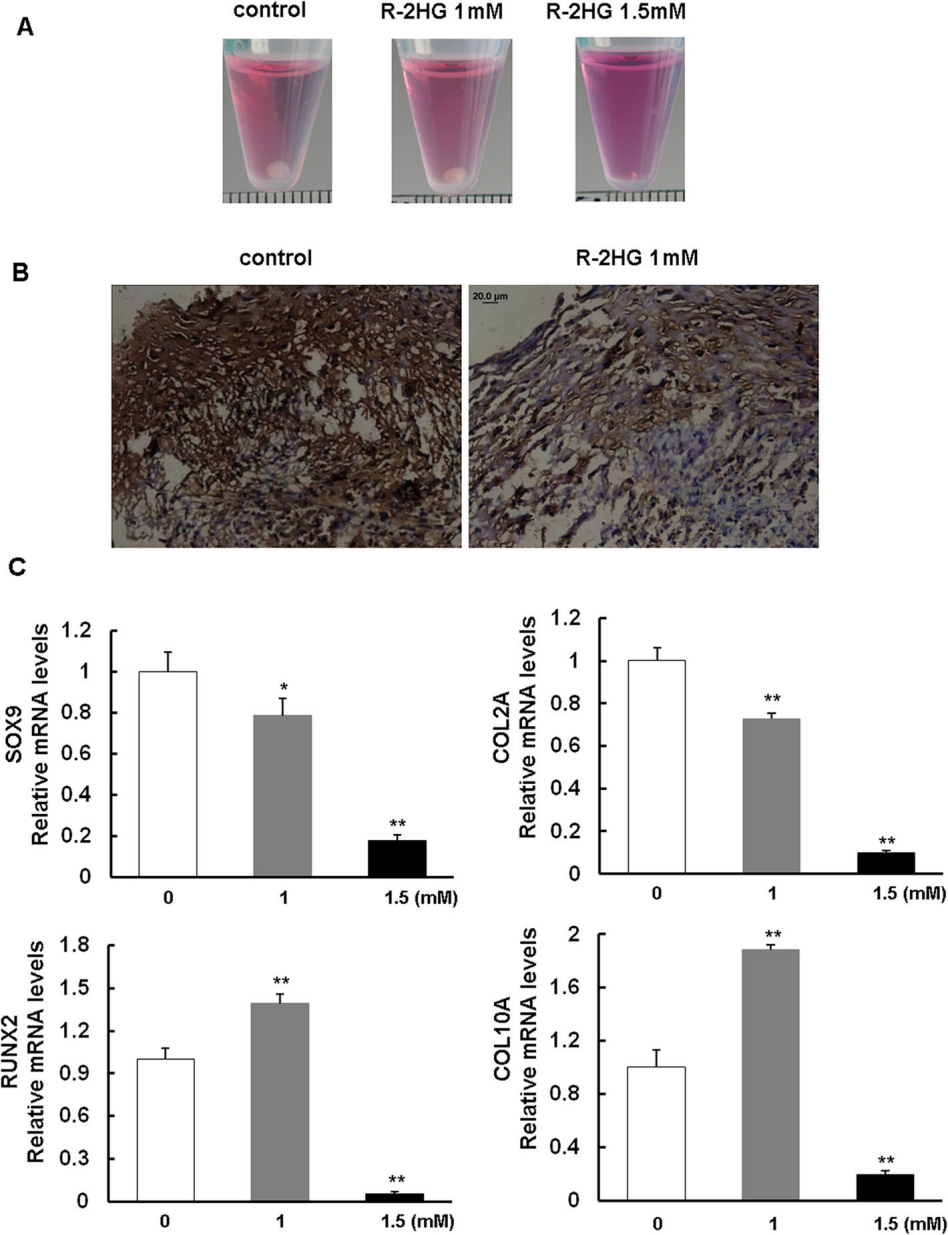
Chondrogenic differentiation of mesenchymal stromal cells (MSCs) in the presence of R-2HG. **a**. Representative photos of pellet formation of MSCs after chondrogenic induction in the absence or presence of R-2HG (1, 1.5 mM). **b**. Collagen 2a staining for pellets in the absence or presence of R-2HG (1 mM). Scale bar = 20 μM (**c)**: Quantitative results of expression levels of differentiation-related genes. * *P <* 0.05 vs the group of MSC in absence of R-2HG. ** *P <* 0.01 vs the group of MSC in absence of R-2HG

To evaluate the effect of R-2HG on chondrogenic differentiation property of MSCs, the cells were made into cell pellets of high-density and then were induced for chondrogenesis for 21 days. As is shown in Fig. 2a, the physical dimension of the pellets in the presence of 1.5 mM R-2HG showed marked decrease compared to those in the absence of R-2HG. Morphologically, matrix deposition as well as collagen 2a (COL2A) staining in cell pellets showed decreased growth in the presence of 1.0 mM R-2HG (Fig. 2b). The pellets of MSCs in the presence of 1.5 mM R-2HG failed to undergo immunohistochemistry. The expression of chondrogenic markers including SOX9 and COL2A1 demonstrated down-regulation in MSCs treated with R-2HG at 1.0 mM and 1.5 mM. However, hypertrophic markers including RUNX2 and COL10A1 were up-regulated in 1.0 mM R-2HG treated group, while down-regulated in 1.5 mM treated group (Fig. 2c). These data confirmed that R-2HG suppresses chondrogenic differentiation of human MSCs, but might promote the onset of chondrocyte hypertrophy in lower concentrations.

### R-2HG promote the adipogenic differentiaion of MSCs

**Fig. 3:**
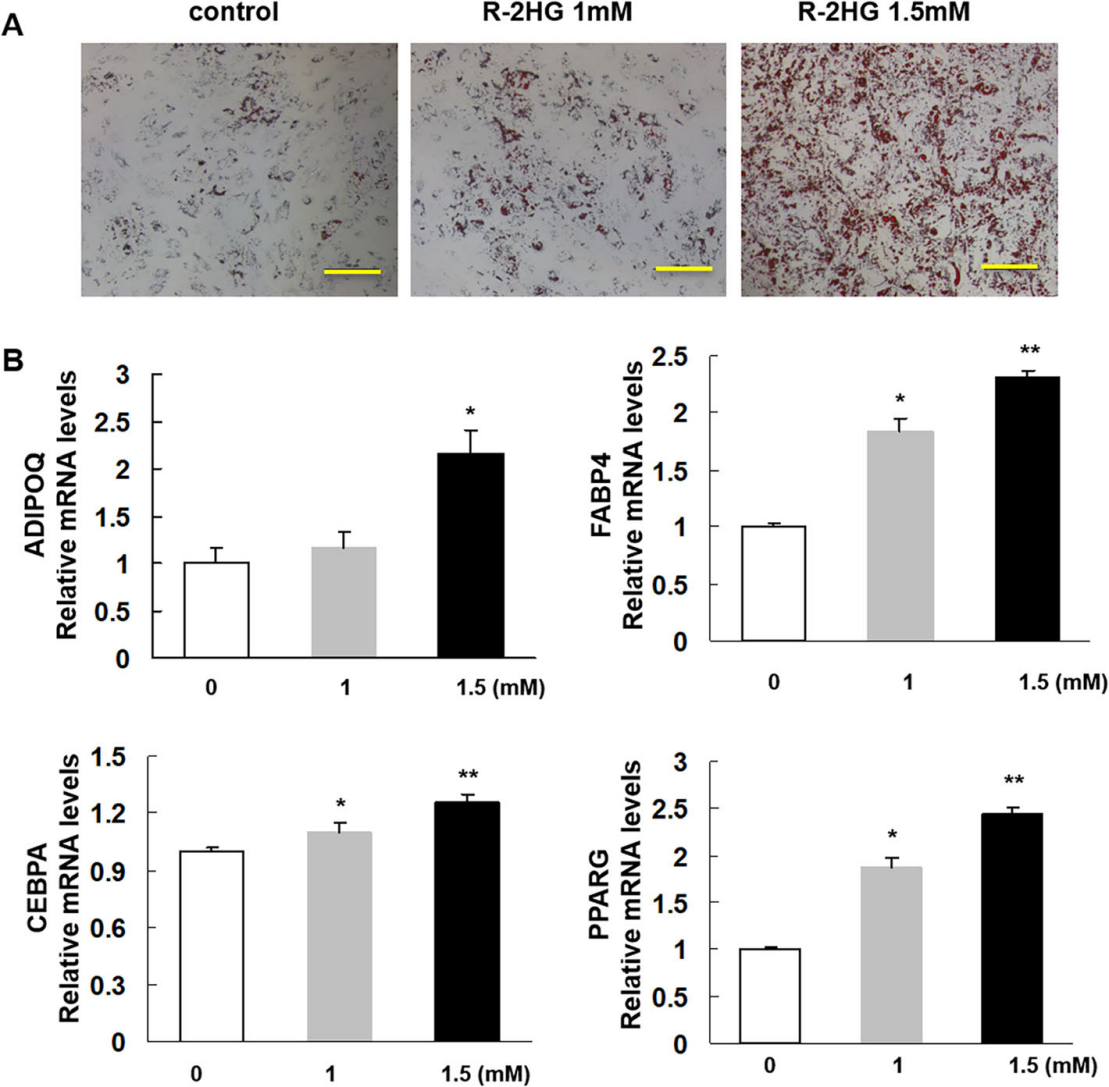
Adipogenic differentiation of mesenchymal stromal cells (MSCs) in the presence of R-2HG. **a**. MSCs were grown in adipogenic differentiation medium for 21 days in the absence or presence of R-2HG (1, 1.5 mM). Lipid vacuoles were assessed by oil red O staining. Scale bar = 100 μM (**b**): Quantitative results of the expression levels of differentiation-related genes. * *P <* 0.05 vs. the group of MSCs in absence of R-2HG. ** *P <* 0.01 vs. the group of MSCs in absence of R-2HG

Next, the effect of R-2HG on adipocytic differentiation was evaluated. MSCs with 100% confluence were induced in adipogenic medium. As is shown in Fig. 3a, R-2HG (at 1 and 1.5 mM) promoted adipogenic differentiation of MSCs which is measured by increased lipid vacuoles (oil red O staining). Furthermore, R-2HG enhanced the relative mRNA expression of adipocyte-specific transcription factors (CEBPA and PPARG) and the marker genes (ADIPOQ and FABP4), supporting the above functional results (Fig. 3b).

### R-2HG induced a pronounced DNA hypermethylation state of MSCs both in proliferating and differentiating conditions

R-2HG affects histone modification and DNA methylation. DNA methylation is considered as a critical epigenetic modification that regulates the differentiation of stem cells, and so the changes in DNA methylation of MSCs exposed to R-2HG were explored.

**Fig. 4:**
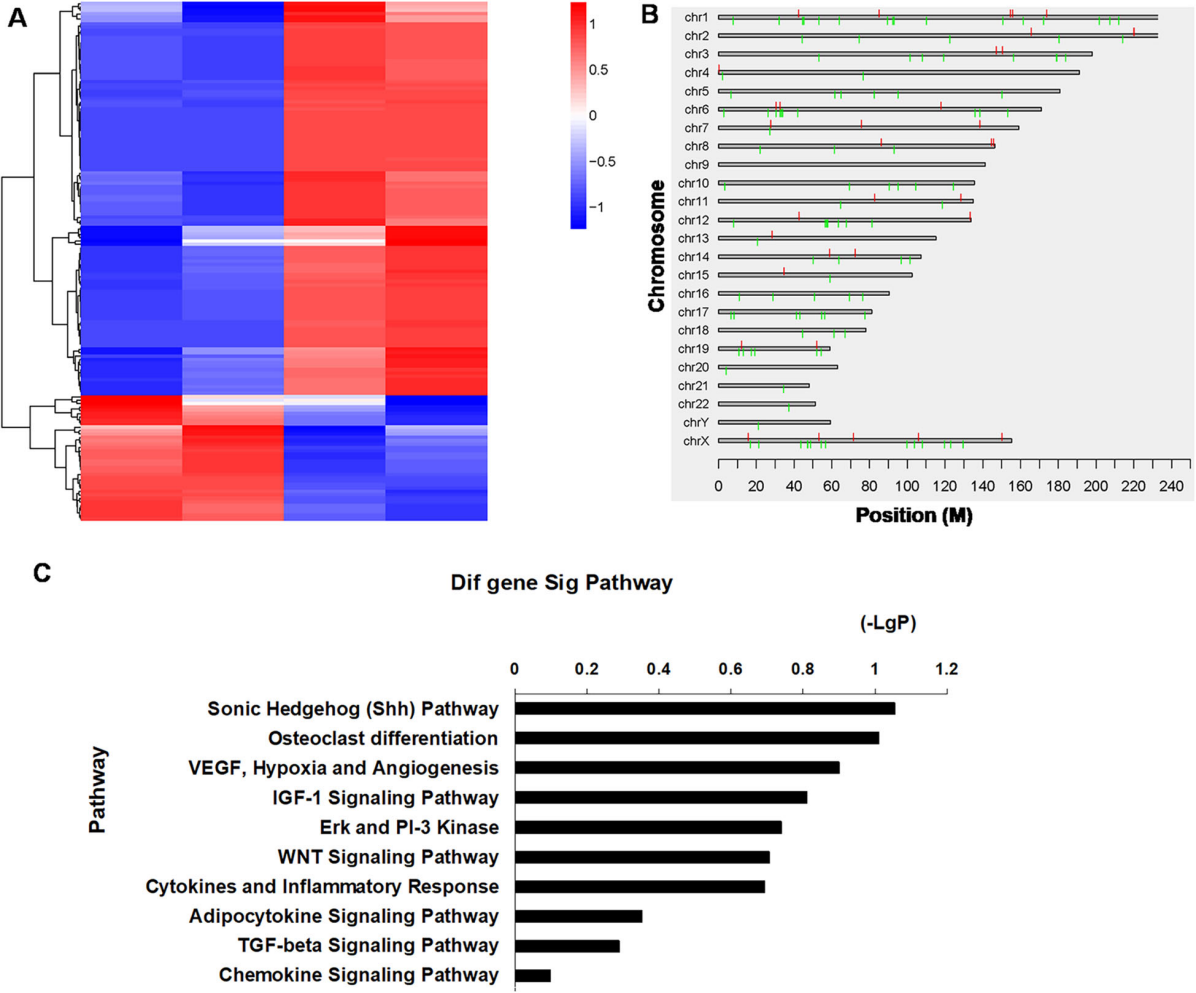
R-2HG induced DNA hypermethylation states of mesenchymal stromal cells (MSCs) in the proliferating condition. **a**: Heatmap displays 154 genes with significant differences in the expression levels between MSCs in the absence or presence of R-2HG (1 mM). **b**: Distribution pattern of DNA methylation. **c**: Results of ingenuity pathway analysis (IPA)

Firstly, we analyzed the DNA methylation of MSCs in proliferation condition. As is shown in Fig. 4a, R-2HG treated MSCs showed a profound DNA hypermethylation at CpG islands when compared with control. 154 differentially methylated CpGs between the two groups were identified. A more detailed analysis of the differential distribution pattern of DNA methylation revealed wide-spread global changes, equally affecting all chromosomes (Fig. 4b). In R-2HG group, hypermethylation was found in 117 genes and hypomethylation in 37 genes (Supplemental Table S1). In addition, the most significantly hypermethylated genes in R-2HG treated samples included stem cell differentiation regulators such as GFI1, GEFT and RUNX1.In order to gain deep insights into the mechanism of aberrant DNA methylation, IPA was performed. The data implicated several signaling pathways involved in MSC differentiation including the Sonic Hedgehog (Shh), insulin/insulin-like growth factor and Wnt signal pathways (Fig. 4c).

**Fig. 5:**
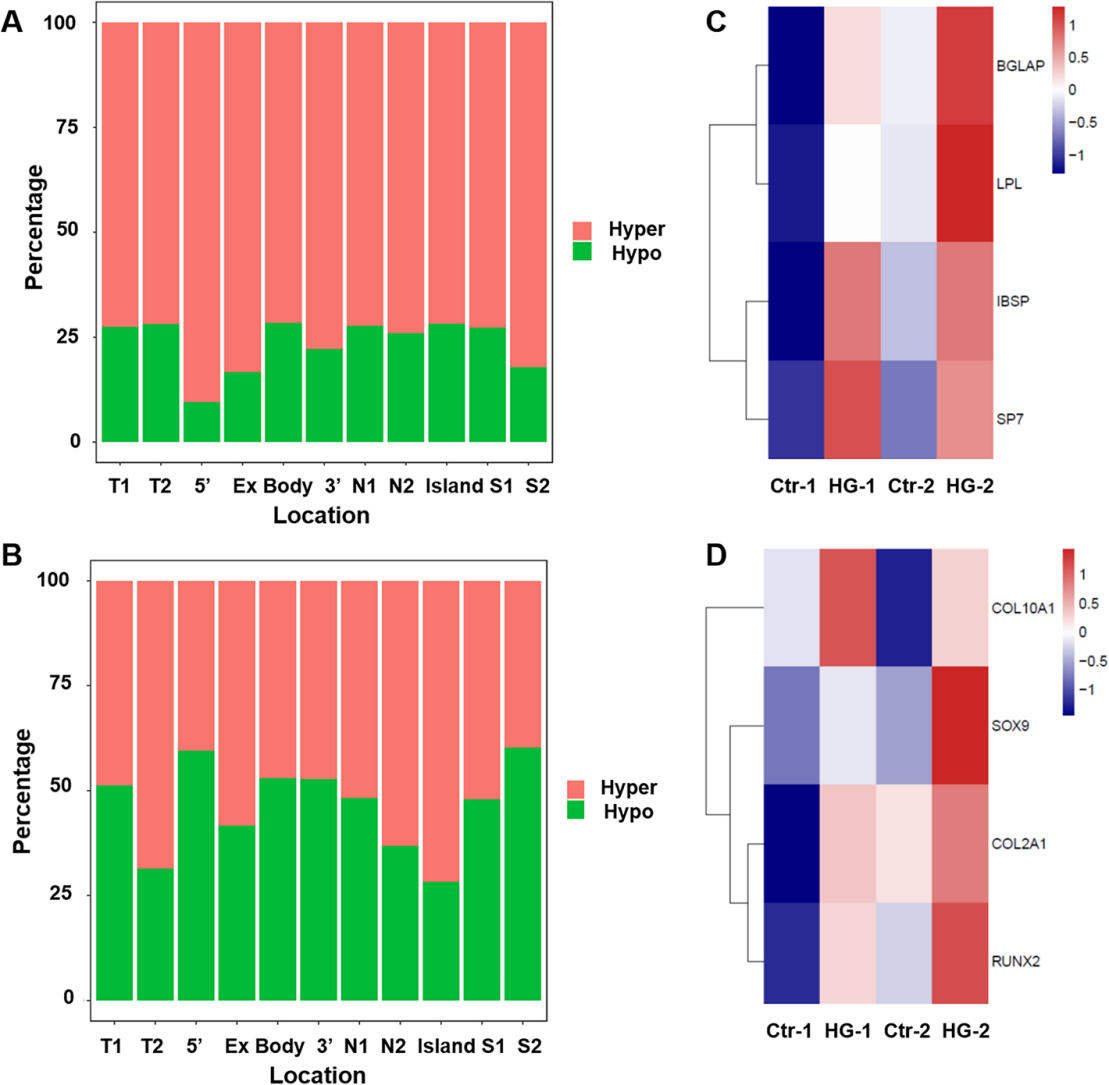
R-2HG induced DNA hypermethylation during mesenchymal stromal cells (MSCs) differentiation. **a**. Stacking bar graph showing percentages of hyper and hypo methylated CpG sites of R-2HG vs. Control MSCs during osteogenic differentiation. **b**. Methylation of lineage-specific genes during osteogenic differentiation. **c**. Percentages of hyper and hypo methylated CpG sites of R-2HG treated MSCs vs. Control MSCs during chondrogenic differentiation. **d**. Methylation of lineage-specific genes during chondrogenic differentiation

Next, we detected the methylation patterns of MSCs during osteogenic and chondrogenic differentiation by array-based assay. In agreement with results from proliferation conditions, R-2HG treated MSCs showed a significantly greater proportion of highly methylated CpG sites during osteogenic differentiation compared to the control (Fig. 5a). The data demonstrated that there were 1462 differentially methylated CpG sites (Δβ < 0.10; *P* < 0.05), including 1098 hypermethylation and 364 hypomethylation sites in the R-2HG treated MSCs compared to control. A more detailed analysis revealed that hypermethylation sites localized in both gene region (including TSS1500, TSS200, 5’UTR, 1stExon, Body and 3’UTR) and CpG context (including N-shelf, N-Shore, CpG islands, S-Shore and S-Shelf). Furthermore, we analyzed the methylation levels of BGLAP, IBSP, LPL, SP7 in R-2HG treated and control MSCs. The results showed that a remarkable hypermethylation of these genes in R-2HG treated group (Fig. 5b).

Intriguingly, the methylation state of chondrogenic differentiation is different. The data showed that there were 1482 differentially methylated CpG sites (Δβ < 0.10; *P <* 0.05), including 788 hypermethylation and 694 hypomethylation sites in the R-2HG group compared to control. Although R-2HG treated MSCs revealed a high ratio of hypermethylation among CpG islands, there was more hypomethylation sites in the 5′ untranslated region and S-shelf (Fig. 5c).

Moreover, the methylation levels of chondrogenic differentiation related genes including SOX9, RUNX2, COL2A1 and COL10A1were analyzed. As shown in Fig. 5d, a higher hypermethylation level of these gene promotors also be found in R-2HG group. Thus, hypermethylation of related genes induced by R-2HG might contribute to the impairment in the differentiation of MSCs.

### R-2HG induced DNA hypermethylation of sonic hedgehog signal components and decreased their expression

As is shown in Fig. 4c, the Shh signaling is considered as the most enriched pathway identified by IPA assay, and regulates cell differentiation. Shh signaling is initiated through the binding of Shh ligand to its transmembrane receptor Ptch1, relieving the suppression of Smo, the transmembrane protein. Smo activates an intracellular cascade that promote the activation of Gli transcription factors (Gli-1, 2, and 3). These Gli family members in turn mediate the transcription of genes controlling cell proliferation, differentiation, and survival [26].

**Fig. 6:**
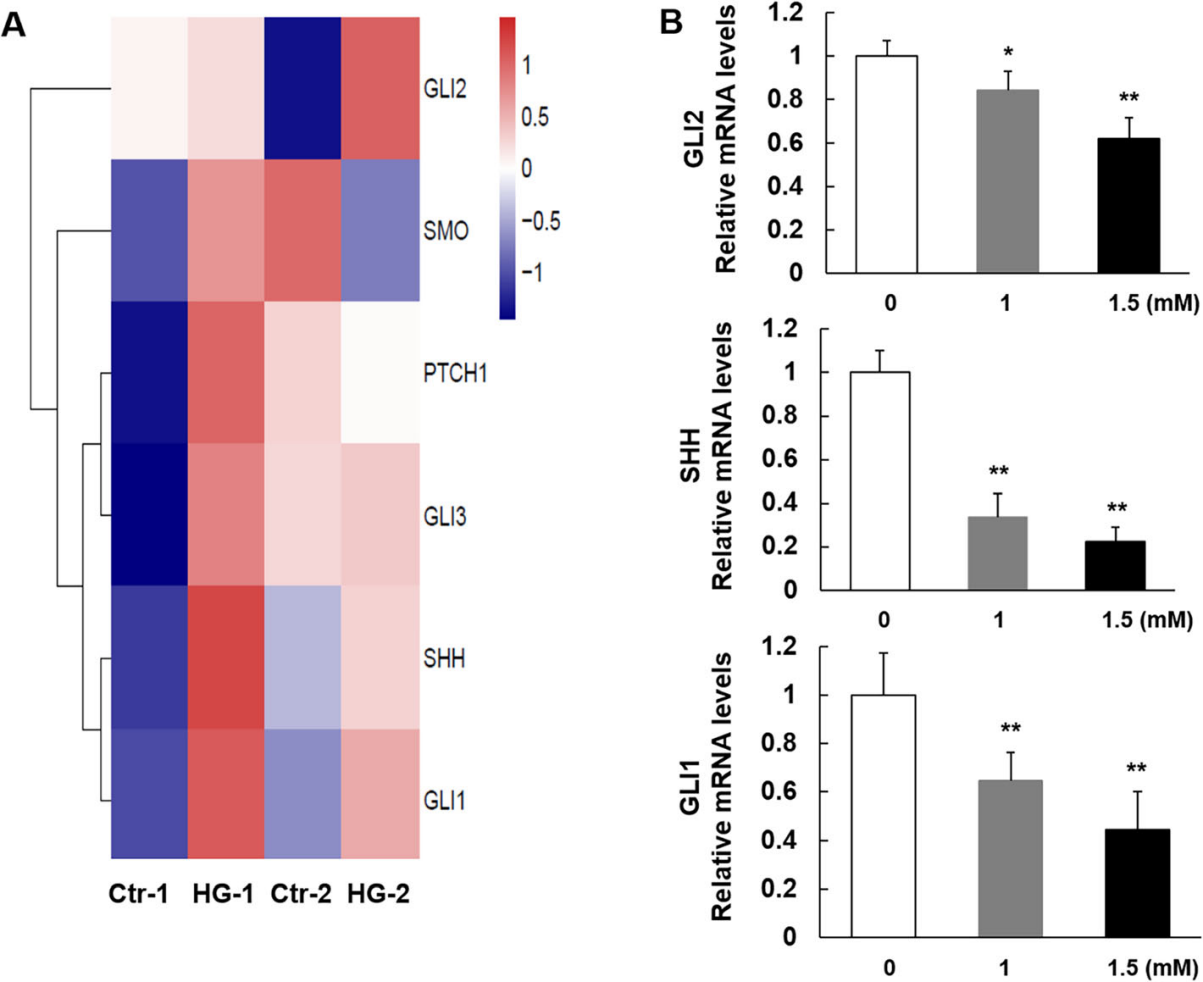
The DNA methylation level and mRNA expression of Sonic Hedgehog signaling components of MSCs exposed to R-2HG during osteogenic differentiation. **a**. Methylation levels of SHH signaling genes in R-2HG treated and control MSCs from two donors. **b**. Expression of SHH signaling genes were detected by quantitative RT-PCR. Data are presented as mean ± S.D. and performed in triplicate from an experiment representative of three independent experiments. *P < 0.05 vs. the group of MSCs in absence of R-2HG. ** *P <* 0.01 vs. the group of MSCs in absence of R-2HG

To validate and further analyze the array-predicted pathway, we analyzed the methylation levels of SHH signaling components including SHH, PTCH1, SMO, GLI-1, 2 and 3 in MSCs during osteogenic differentiation. The results showed that a significant hypermethylation of SHH, GLI-1 and GLI-2 in R-2HG treated cells (Fig. 6a). However, the methylation states of PTCH1, SMO and GLI-3 were inconsistent between two donors derived MSCs.

Furthermore, the mRNA expression of SHH pathway component genes were quantified by RT-PCR. Our data showed that SHH, a secreted glycoprotein that activates Shh pathway, decreased in a dose-dependent manner in MSCs treated by R-2HG (Fig. 6b). Moreover, GLI1 and GLI2, key markers of Shh signaling, were both down-regulated significantly in R-2HG group (*p* < 0.01). However, no changes in the expression levels of PTCH1, Gli3 or SMO were detected.

These results revealed that the oncometabolite R-2HG induced by mutant IDH mutation blocked the osteogenic and chongenetic differentiation, while promoted the adipogenic differentiaion of MSCs. The underlying mechanisms might be associated with hypermethylation of stem cell differentiation related genes, such as Shh signaling.

## Discussion

Some metabolites play a critical role as regulators of some important enzymes in various biological pathways. According to recent studies, metabolic alterations promote the initiation and development of malignant cells. R-2HG that is produced by mutant IDH proteins is regarded as a prototype of these oncometabolites, and a serious of studies have proved the role of R-2HG in malignant transformation [13, 27]. Elevated levels of R-2HG that are caused due to mutations in IDH1 and IDH2 are frequently shown (up to 87%) in enchondromas [4]. Impaired differentiation by R-2HG has been reported in central nervous system and during hematopoietic differentiation processes [13, 27]. We therefore examined the effects of R-2HG on the characteristics, especially on the differentiation properties of human MSCs, which presumably act as precursors of cartilaginous tumors.

The results of the present study showed that R-2HG impaired the calcification of MSCs and reduced the expression of both early and late osteoblast differentiation-related genes in a dose-dependent manner, indicating the inhibition of osteogenic differentiation of MSCs by R-2HG. In consistent with our data, Suijker et al. [28] recently reported impaired development of vertebrate rings in zebrafish with the presence of R-2HG, suggesting that R-2HG blocks osteoblast differentiation in vivo. Interestingly, the results of our study along with the previous study [28] demonstrated that R-2HG inhibited osteogenic differentiation, while mutations are virtually rare in osteosarcoma and very frequently found in cartilaginous tumors [4].

We next investigated the effect of R-2HG on chondrogenic differentiation of MSCs. The results indicated that R-2HG suppressed chondrogenic differentiation of human MSCs, but might promote the onset of chondrocyte hypertrophy at lower concentration (1.0 mM). Lu et al. [11] demonstrated that the expression of mutant IDH2 in 3 T3-L1 cells results in a profound impairment in chondrocyte differentiation and was consistent with our study results. Recently, Hirata et al. [29] reported dysregulation of chondrogenic differentiation with persistence of hypertrophic chondrocytes from mice with the mutant IDH1 or control chondrocytes treated with R-2HG, preventing the bone from normal replacement of cartilage. However, Suijker et al. [28] indicated that half of the MSCs showed an increase in differentiation towards chondrogenic lineage in the presence of R-2HG. Variations in these results might occur due to differences in concentrations of R-2HG used in the experiments. In this study, hypertrophic markers including Runx2 and Col10a were up-regulated under chondrogenic differentiation conditions in the presence of 1.0 mM R-2HG. Runx2 is an important transcription factor in chondrocyte hypertrophy that promotes the expressions of Col 10, thus disturbing chondrocyte homeostasis [30].

Compelling evidences indicated that IDH1/2 mutation is sufficient to initiate enchondromas and sarcomas in vivo [11, 29]. MSCs give rise to variations in differentiated cells, including adipocytes, osteocytes, neural cells, stromal cells, chondrocytes, muscle cells and fibroblasts,, which are thought to be the progenitor cells of many different types of sarcomas [23]. Our data indicated that increased levels of R-2HG blocked osteogenic differentiation and disturbed the normal chondrogenic differentiation of MSCs, partly explaining the mechanism of cartilage tumor formation induced by IDH mutation. The mechanisms of tumorigenesis is therefore comparable to other tumors caused by IDH mutations, as the differentiation was impaired in hematopoietic precursor cells [17], neurogenic precursor cells [18] and liver [31] progenitor cells.

Besides osteogenic and chondroblastic differentiation, MSCs is also able to differentiate into adipocytes. We herein showed that R-2HG promoted adipogenic differentiation of MSCs as measured by increased lipid vacuoles and enhanced gene marker expression. This was opposite to the reduced adipogenic differentiation caused by R-2HG or by introduction of an IDH2 mutation in 3 T3-L1 cells [11]. These murine 3 T3-L1 cells involve spontaneous adipogenic differentiation. The effect on human MSCs was studied to explain the differences in the results. MSCs are delicately balanced during their adipo-osteogenic differentiation. Many in vitro investigations have proved that fat-induction factors inhibited osteogenesis, and in contrast, the bone-induction factors hindered adipogenesis [32]. Our results also confirmed that R-2HG inhibited osteogenesis, while promoted the adipogenesis of MSCs.

A key issue in IDH1/2 mutation-induced tumorigenesis is the blockage of cellular differentiation [18]. Though the precise oncogenic consequences of IDH mutations remained unclear, high levels of R-2HG is widely believed to be essential in the process. R-2HG competitively inhibited multiple a-KG-dependent dioxygenases, including key epigenetic regulators, histone demethylases and DNA-demethylating agents for example [10]. According to a previous study, CpG island methylation was found increased significantly in IDH mutant chondrosarcoma samples [11]. In addition, Jin et al. recently showed that IDH1 R132C mutation increased histone methylation in both cartilage- and bone-related genes and global histone methylation [33]. However, the effects of R-2HG on DNA methylation status of MSCs are still unknown.

We herein showed that R-2HG induced a pronounced DNA hypermethylation state of MSCs both in proliferation and osteogenic differentiation conditions. R-2HG treated MSCs revealed a high ratio of hypermethylation among CpG islands during chondrogenic differentiation, however, there were more hypomethylation sites in some gene regions. The varies of methylation status might be result from the different induction condition, such as a higher concentration of ascorbic acid, which induces TET-dependent DNA demethylation [34]. Our data further confirmed that active DNA methylation does occur on lineage-specific gene promotors during R-2HG induced osteogenic and chondrogenic differentiation. DNA hypermethylation is regarded as a barrier in the differentiation of MSCs [35]. Thus, hypermethylation of related genes induced by R-2HG might contribute to the impairment in the differentiation of MSCs.

In functional analysis, the top canonical pathway is the Shh signaling. Previous studies indicated that increased Shh signaling promoted osteogenesis in various bone-forming cells, and in contrast, Shh signaling repressed adipogenic differentiation in preadipocytes [36, 37]. In addition, Shh promoted chondrogenesis in MSCs by inducing the expression of Sox9 [38]. A recent study indicated that mechanical stimulation promoted osteogenic differentiation of MSCs through epigenetic regulation of Shh [39]. Moreover, Shh and Gli genes play an essential role during cartilage development [40]. Our results showed that R-2HG impaired the osteogenic differentiation of MSCs, which in turn was accompanied by down-regulation of Shh signaling, implying that Shh signaling might play a role in this process.

Due to lack of effective treatment strategies for advanced diseases, the clinical management of chondrosarcomas remains exceptionally challenging [41]. Somatic mutations of IDH genes exist in more than 50% of primary conventional chondrosarcomas. More recently, the first mutant IDH2 inhibitor, enasidenib (AG-221), in patients with relapsed or refractory IDH2-mutated AML has been approved by FDA [42]. The development of IDH inhibitors is an emerging treatment option for patients with chondrosarcoma.

## Conclusion

In conclusion, the present study results showed that R-2HG impaired osteogenic and chondrogenic differentiation of MSCs possibly via inducing DNA hypermethylation. These results provide novel insights into the role of R-2HG in the development of cartilaginous tumors.

## Supplementary Information

**Additional file 1: Supplement Fig. 1.** Proliferation and phenotype of mesenchymal stromal cells (MSCs) in the presence of R-2HG. A. Proliferation of MSCs in the absence or presence of R-2HG (0.1–1.5 mM). B. Immunophenotype of MSCs in the absence or presence of R-2HG (1.5 mM) was determined by flow cytometry. Red lines represent the fluorescence intensity histograms (FIH) with isotype control. Blue lines represent FIH for membrane antigen of MSCs in the absence of R-2HG. Black lines represent the FIH for membrane antigen of MSCs in the presence of R-2HG (1.5 mM).

**Additional file 2.**

## Data Availability

All data generated or analyzed during this study are available upon request.

## Abbreviations

IDH: Isocitrate dehydrogenase
R-2HG: R-enantiomer of 2-hydroxylglutarate
MSCs: Mesenchymal stromal cells
Shh: Sonic Hedgehog
α-KG: α-ketoglutarate
BM: Bone marrow
FBS: Fetal bovine serum
PBS: Phosphate buffered saline
LG-DMEM: Low-glucose Dulbecco’s modified Eagle’s medium
CCK-8: Cell Counting Kit-8 assay
RT-PCR: Real-time quantitative polymerase chain reaction analysis
GAPDH: Glyceraldehydes 3-phosphate dehydrogenase
IPA: Ingenuity Pathway Analysis
