# Supplementary figures and images for: The oncometabolite R-2-hydroxyglutarate dysregulates the differentiation of human mesenchymal stromal cells via inducing DNA hypermethylation
Source: BMC Cancer. 2021 Jan 7;21:36. doi: 10.1186/s12885-020-07744-x (PMC7791852; doi:10.1186/s12885-020-07744-x)

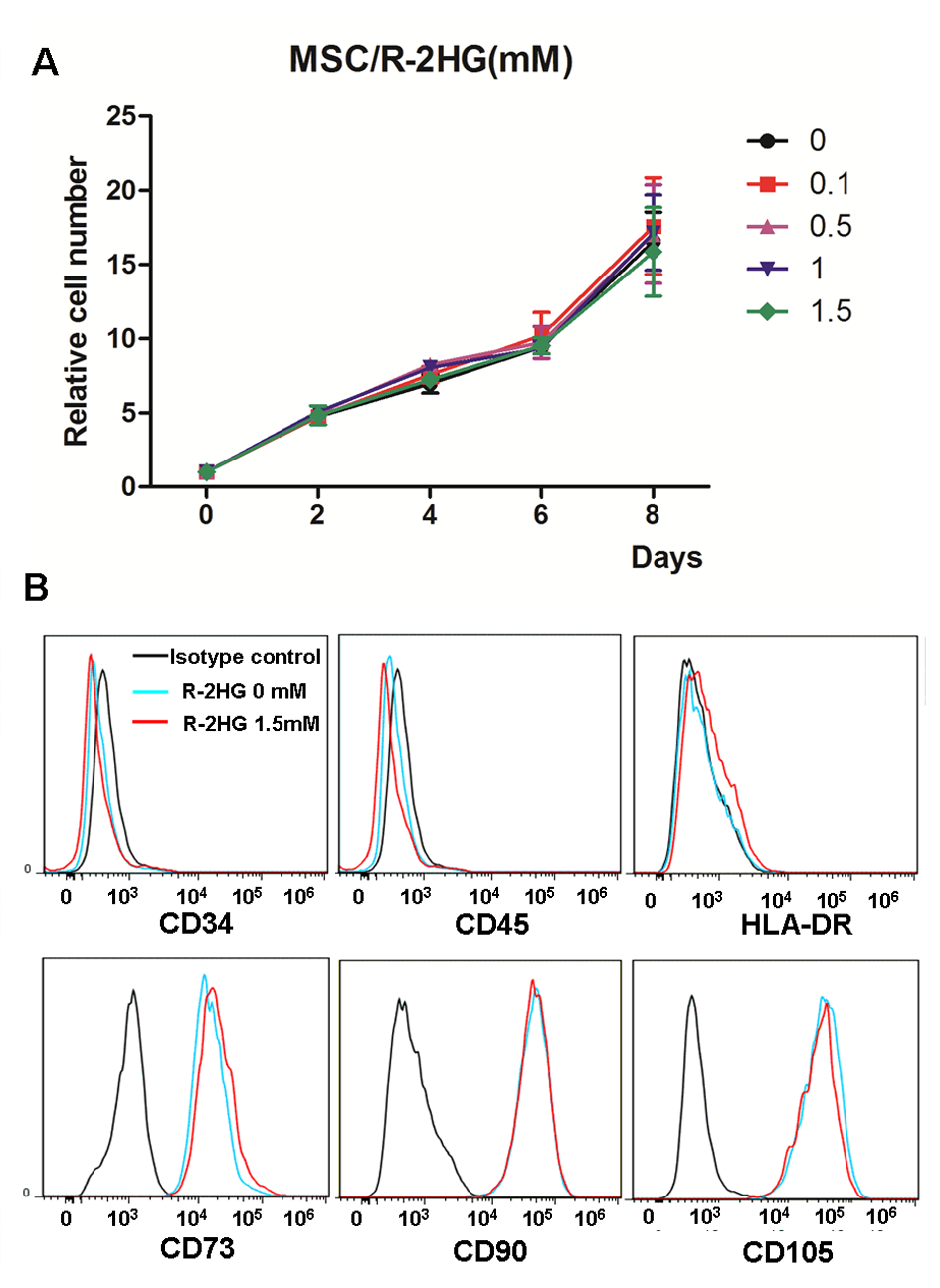

Supplement: Supplementary file 1 — Additional file 1: Supplement Fig. 1. Proliferation and phenotype of mesenchymal stromal cells (MSCs) in the presence of R-2HG. A. Proliferation of MSCs in the absence or presence of R-2HG (0.1–1.5 mM). B. Immunophenotype of MSCs in the absence or presence of R-2HG (1.5 mM) was determined by flow cytometry. Red lines represent the fluorescence intensity histograms (FIH) with isotype control. Blue lines represent FIH for membrane antigen of MSCs in the absence of R-2HG. Black lines represent the FIH for membrane antigen of MSCs in the presence of R-2HG (1.5 mM). [file 12885_2020_7744_MOESM1_ESM.tif]
